# Time trends of baseline demographics and clinical characteristics of HIV infected children enrolled in care and treatment service in Dar es Salaam, Tanzania

**DOI:** 10.1186/s12879-015-0875-2

**Published:** 2015-03-26

**Authors:** David Sando, Donna Spiegelman, Lameck Machumi, Mary Mwanyika-Sando, Eric Aris, Aisa Muya, Elizabeth Jackson, Till Baernighausen, Ellen Hertzmark, Guerino Chalamilla, Wafaie Fawzi

**Affiliations:** Management and Development for Health (MDH), P.O. Box 79810, Dar es Salaam, Tanzania; Harvard T.H. Chan School of Public Health (HSPH), P.O. Box 79810, Dar es Salaam, Tanzania

**Keywords:** HIV, AIDS, Pediatric

## Abstract

**Background:**

Few studies have described time-based trends of clinical and demographic characteristics of children enrolling in HIV and AIDS care and treatment services. We present findings of a study that explored time-based trends of baseline characteristics among children enrolling into 26 public HIV care facilities in Dar es Salaam, Tanzania.

**Methods:**

Children enrolled between October 2004 and September 2011 was included in these analyses. The year of enrollment was used as the primary predictor of interest, and log linear and linear regressions model were used to analyze dichotomous and continuous variables respectively. P-values under 0.05 were considered significant.

**Results:**

Among the 6,579 children enrolled, the proportion with advanced disease at enrollment increased from 35% to 58%, mean age increasing from 5.0 to 6.2 years (p < 0.0001), proportion of children less than 2 years decreased from 35% to 29%. While the median hemoglobin concentration rose from 9.1 g/dl to 10.3 g/dl (P <0.0001), proportion with a history of past TB dropped from 25% to 12.8% (P < 0.0001). Over time, health centers and dispensaries enrolled more children as compared to hospitals (P < 0.0001). Temeke district, which has the lowest socioeconomic status among the three districts in Dar es Salaam, had a significant increase in enrollment from 22% to 25% (P = 0.02).

**Conclusion:**

We found that as time progressed, children were enrolled in care and treatment services at an older age sicker status as evidenced by increase in mean age and more advanced disease stage at first contact with providers. We recommend more efforts be focused on scaling up early HIV infant diagnosis and enrollment to HIV care and treatment.

## Background

The public provision of free pediatric antiretroviral treatment (ART) services in Tanzania started in 2004. Country-wide HIV and AIDS data show that the proportion of eligible children receiving highly active antiretroviral therapy (HAART) has remained relatively constant over the past 5 years at about 25% [1]. Despite revisions of national pediatric ART guidelines to extend eligibility to all confirmed HIV infected children younger than 2 years of age, irrespective of CD4 cell count, CD4 percentage, or WHO pediatric stage, approximately 76% of ART eligible children have yet to access HAART services [2,3].

Poor coverage and utilization of ART services has serious repercussions, especially for children living with HIV. Approximately 50% of HIV infected children not initiated on ART die before the age of 2 years, and of the surviving, about one third die before 5 years of age [4,5]. Early initiation of HAART leads to a significant decline of early mortality [5-7], with up to 90% survival of children up to age 5 [5,6,8]. In Rakai, Uganda, HIV infected children were found to have high two year mortality of 547 per 1000 as compared to 166 per 1000 of HIV negative children of HIV positive mothers, and 128 per 1000 of HIV negative mothers [6]. The 2012 Tanzania Ministry of Health and Social Welfare (MOHSW) report shows that only 56% of children living with HIV are enrolled in pediatric HIV care and monitoring services [9]. The observed low enrollment may be attributed to several factors including poor access to early infant diagnosis of HIV (EID); insufficient number of trained clinicians in the management of pediatric HIV/AIDS; limited health care infrastructure; socio-cultural factors; lack of affordable simple diagnostic HIV testing technologies for children less than 18 months; limited experience with simplified standardized treatment guidelines; and lack of affordable practicable pediatric antiretroviral (ARV) formulations, which further complicates the scaling up of the national pediatric ART program [4,10,11].

The rapid disease progression and high mortality, which may occur in untreated children, raises the need for urgent scaling up of the ART program to bolster access and coverage [11-13]. Sub-optimal coverage and poor retention for patients who are on HAART exposes the need for robust evaluations of pediatric ART services. Very few analyses have described trends in characteristics of children at enrollment of HIV care and treatment programs [14-16]. This information is essential to understanding the progress achieved thus far in increasing enrollment, as well as in designing strategies aiming to improve access to HAART for the large number of HIV infected and untreated children.

Since 2004, Management and Development for Health (MDH) (and its predecessor) has provided technical support to the implementation of pediatric HIV care and treatment services through the President’s Emergency Plan for AIDS Relief (PEPFAR). This program was rolled out in a wide range of public and private health care facilities in Dar es Salaam, Tanzania. Despite a rapid increase in the number of HIV-positive patients enrolled in care and treatment since the inception of the program, children continue to be under-represented. This study describes temporal trends in baseline demographic and clinical characteristics amongst children enrolled in the ART program in 26 care and treatment clinics (CTCs) in Dar es Salaam.

## Methods

### Study population

Between October 2004 and September 2011, 6579 children under 15 years of age were enrolled in the national ART program in 26 CTCs located in the Dar es Salaam region. The facilities include five secondary level hospitals and 21 primary health care (PHC) clinics that are dispersed within the 3 districts of Dar es Salaam. All facilities offered free health care for HIV infected adults and children. Children who were enrolled in HIV Care and Treatment, both those started on ART and in care for monitoring were included in this analysis. Since the start of the ART program in September 2004 to September 2011, there has been no change in pediatric ART regimens. All enrolled children below 3 years were receiving Zidovudine (AZT) + Lamivudine (3TC) + Nevirapine (NVP) while those eligible and 3 years or above were receiving Zidovudine (AZT) + Lamivudine (3TC) + Nevirapine (NVP)/EFAVIRENZ (EFV) regimen.

### Data collection

The design of the study is a retrospective analysis using routine collected data. At the enrollment visit demographic characteristics were recorded including age, sex, marital status of caretakers and past therapeutic and clinical history. Children were also screened for tuberculosis (TB) symptoms using standardized questionnaire to identify TB suspect, who then took a Acid Fast Bacilli (AFB) test for their sputum, or chest x-ray. History of diarrheal disease for both acute and chronic episodes was documented. Investigation for anemia, assessment of functional status, nutritional status and WHO clinical stage of the disease were also done. However wasting syndrome was not collected for year 2011 due to change of the tools. Immunological and biochemical tests were performed to evaluate CD4, alanine aminotransferase (ALT), and hemoglobin (HB) levels.

### Study variables

Time trends of clinical and demographic variables with programmatic implications were examined. The variables were: sex, age at enrollment (<2, 2–10, >10 years), WHO clinical stage at enrollment (categorized as advanced disease (stages III and IV) or moderate disease (stages I and II)), CD4 cell count for those ≥ 5 years, CD4 cell percentage for those <5 years, past history and current status of TB disease, HB level and presence of OIs. Diarrhea, wasting syndrome, season of the year, facility level, and district were also included. Due to fewer children’s enrolled during the end of 2004 at the start of the program, 2004 enrollment is combined with 2005 in the analysis.

### Statistical methods

Categorical variables are presented in the tables as number (percent), and continuous variables are presented as median (interquartile range) or mean (standard deviation). In all analyses, year of enrollment was used as the predictor. All dichotomous variables were analyzed using log-binomial regression. These included WHO clinical stage, past history of TB, current TB symptoms, presence of OI’s and diarrhea. Continuous characteristics were modeled using linear regression including age, body mass index (BMI) z-score, weight-for-length (WFL) z-score and HB level. To assess the statistical significance of time trends with nominal categorical variables with more than two levels, such as district, season of enrollment and level of the health facility, we used nominal polytomous logistic regression. Stepwise restricted cubic splines [17] were used to assess non-linearity of time trends in relation to dichotomous and continuous outcome of interest. P-values under 0.05 were considered significant.

This study project received IRB approval from the National Institute of Medical Research (NIMR) in Tanzania. A written consent was obtained from parents or guardians of the children involved in this study.

## Results

Table 1 presents the characteristics of 6579 children enrolled in 26 public CTCs in Dar es Salaam. Forty-nine percent were male. The mean age was 5.2 years (sd: 4.2). Thirty-seven percent were less than 2 years old and 20% were 10 or more years old at enrollment. Fifty-one percent presented with advanced WHO pediatric stage (III & IV), and 30% were wasted. For the 49% of the children enrolled at age less than 5 years, their median CD4 percentage was 18 (IQR: 12–26); for those enrolled at age 5 or above, their median CD4 count was 330 cells/mm3 (IQR: 125–586). Fourteen percent had past medical history of pulmonary TB, and 6.4% presented with TB at their first visit to the clinic. The median HB concentration was 9.8 g/dl (IQR: 8.6-11.0), and 14% of the children had at least one type of OI at enrollment. Approximately 4% presented with signs of wasting syndrome and 10% had diarrheal disease. Fifty-five percent of the children were enrolled in secondary health care facilities and 45% of total enrollment occurred in Ilala district.

**Table 1:** **Baseline characteristics of the pediatric patients enrolled in care and treatment clinics**

	**N (%) or median (IQR)**
Number of Children	6579
Proportion children among enrollee	14
Male, n (%)	3209 (48.78)
Age, Mean (sd)	5.2 (4.2)
Age ≥10 years, n (%)	1284 (19.52)
Age < 2 years, n (%)	2416 (37.23)
WHO clinical stages III & IV, n (%)	2937 (50.61)
Proportion with wasting at enrollment (n = 6806)	1109 (29.93)
CD4 cell %, median (IQR) (<5 yrs) n = 3219	18 (12–26)
CD4 cell count (≥5 yrs) n = 3271	330 (125–586)
Proportion with TB history, n (%)	730 (14.54)
Proportion with TB current, n (%)	333 (6.38)
Hemoglobin, median (IQR)	9.8 (8.6-11)
Proportion with diarrhea, n (%)	542 (10.21)
Proportion with OI	894(13.59)
District of enrollment, n (%)	
Ilala	2942 (45.14)
Kinondoni	1909 (29.29)
Temeke	1667 (25.58)
Season of the year	
December - March	1070 (16.26)
April - May	1091 (16.58)
June - September	2070 (31.46)
October - November	2348 (35.69)
Level of health facility, n (%)	
Hospital	3600 (55.23)
Health Center	2549 (39.11)
Dispensary	369 (5.66)
Year of enrollment, n (%)	
2004/05	611 (9.29)
2006	1057 (16.07)
2007	1094 (16.63)
2008	1278 (19.43)
2009	1091 (16.58)
2010	888 (13.50)
2011	560 (8.51)

Table 2 presents demographic and clinical characteristics by year of children enrolled between October 2004 and September 2011. The number of new children enrolled increased from 2004/05 to 2008 then started to decrease in 2009. The mean age at enrollment increased from 5.0 (3.9) in 2004/05 to 6.2 years (std: 4.2: P = <0.0001) in 2011. The proportion of children enrolled at the age of 10 years or above increased from 16% in 2004/5 to 27% in 2011 (P <0.0001), and the fraction of children enrolled at the age of 2 years or less decreased from 35% in 2005 to 29% in 2011 (P = <0.0001). The proportion of children enrolled in Temeke district, which has the lowest socioeconomic status of the three districts, rose from 22% in 2004/5 to 25% in 2011 (P = 0.02). The proportion of children seen in health centers and dispensaries, as compared to the hospitals, increased over the 7 years during the follow up period and was significant (p <0.0001).

**Table 2:** **Characteristics of children enrolled in care and treatment services by Calendar year**

	**Year of enrollment to Care and treatment**							
	**2004/05**	**2006**	**2007**	**2008**	**2009**	**2010**	**2011**	***P-value***
Average number of children enrolled monthly	43.6(7.8)	88.1 (15.8)	91.2 (16.4)	106.5 (19.1)	90.9 (16.3)	74.0 (13.3)	62.2 (11.2)	0.01
Percent of children among total enrollees (total/% children)	(3394) 0.18	(5872) 0.18	(9116) 0.12	(18257) 0.07	(18183) 0.06	(17760) 0.05	(14000) 0.04	<0.01
Male, n (%)	286 (46.8)	501 (47.4)	541 (49.5)	643 (50.3)	532 (48.8)	427 (48.09)	279 (49.8)	0.38
Age, Mean (std)	5.0 (3.9)	3.7 (4.1)	5.5 (4.0)	5.2 (4.2)	5.5 (4.3)	5.7 (4.2)	6.2 (4.3)	<0.01
Age ≥10 years, n (%)	99 (16.3)	133 (12.8)	209 (19.7)	242 (19.1)	240 (22)	216 (24.60)	145 (26.8)	<0.01
Age < 2 years, n (%)	212 (34.9)	574 (55.1)	357 (33.6)	457 (35.9)	380 (34.9)	279 (31.78)	157 (29.)	<0.01
WHO clinical stages III & IV, n (%)	122 (34.9)	306 (45.3)	538 (53.4)	649 (51.5)	556 (51)	446 (50.86)	320 (57.87)	0.03
Proportion with wasting	8 (33.3)	22 (39.3)	235 (34.4)	327 (29.3)	284 (27.6)	233 (29.4)	--	0.01
CD4 cell % (<5 yrs), median (IQR)	16 (9-2)	21 (12-31)	16 (11-21)	17 (12-3)	18 (13-3)	19 (12-26)	18 (14-25)	0.89
CD4 cell count (≥ 5 yrs)	293 (90-574)	320 (122-610)	317 (130-543)	333 (121-565)	357 (134-601)	349 (129-636)	3322 (121-631)	0.09
Proportion with TB history, n (%)	98 (25.32)	38 (14.2)	148 (19.2)	157 (13.4)	131 (12.4)	92 (10.9)	66 (11.8)	<0.01
Percent with TB symptoms, n (%)	57 (13.8)	13 (3.9)	36 (4.2)	75 (6.3)	62 (5.8)	52 (6.1)	38 (6.8)	0.07
Hemoglobin, median (IQR)	9.1 (8.1-10.4)	9.4 (8.4-10.7)	9.5 (8.3-10.6)	9.8 (8.6-11)	9.8 (8.7-11)	10.2 (9-11)	10.3 (9-11)	<0.01
Proportion with OI, n (%)	48 (7.9)	28 (2.7)	163 (14.9)	247 (19.3)	185 (16.9)	122 (13.7)	101 (18.)	<0.01
Proportion with diarrhea, n (%)	67 (15.5)	38 (11.4)	91 (10.5)	142 (11.8)	107 (9.9)	48 (5.6)	49 (9.2)	<0.01
District of enrollment, n (%)								
Ilala	252 (42.5)	489 (47.6)	528 (48.8)	580 (45.4)	493 (45.2)	363 (40.9)	237 (42.3)	0.02
Kinondoni	210 (35.4)	298 (29)	301 (27.8)	321 (25.1)	307 (28.1)	289 (32.6)	183 (32.7)	
Temeke	131 (22.1)	240 (23.4)	252 (23.3)	377(29.5)	291 (26.7)	236 (26.6)	140 (3)	
Season of enrollment								
December - March	181 (29.6)	269 (25.5)	333 (30.4)	362 (28.3)	404 (37)	297 (33.5)	224 (4?)	<0.01
April - May	62 (10.2)	191 (18.1)	157 (14.4)	240 (18.8)	177 (16.2)	163 (18.4)	80 (14.3)	
June - September	254 (41.6)	366 (34.6)	402 (36.8)	450 (35.2)	349 (31.9)	272 (30.6)	256 (10.9?)	
October - November	114 (18.7)	231 (21.9)	202 (18.5)	226 (17.7)	162 (12.7)	156 (17.6)		
Level of health facility, n (%)								
Hospital	486 (81.9)	706 (68.7)	611 (56.5)	674 (52.7)	526 (48.2)	391 (44.)	206 (36.8)	<0.01
Health Center	95 (16.)	317 (30.9)	470 (43.5)	585 (45.8)	455 (41.7)	371 (41.8)	256 (45.7)	
Dispensary	12 (2)	4 (0.4)	0 (0)	19 (1.5)	110 (10.)	126 (14.19)	98 (17.50)	

We also observed an increase in children presenting with advanced clinical stage (WHO pediatric stage III and IV) from 35% in 2004/5 to 58% in 2011 (p = 0.03). There was a decrease in the percent of children presenting with a history of TB disease from 25% in 2004/5 to 13% in 2011 (p <0.0001). The median HB level increased from 9.1 mg/dl (IQR: 8.1-10.4) in 2004/5 to 10.3 mg/dl (IQR: 9.4-11.3) in 2011 (p < 0.0001). There was a decrease in the proportion of children with diarrhea from16% in 2004/5 to 9% in 2011 (p < 0.0001). However, consonant with the increase in advanced disease, there was an increase in the proportion of children with OI’s from 8% in 2004/5 to 18% in 2011 (p < 0.0001). There was no significant non-linearity evident in any of the time trends studied.

## Discussion

This study is among the few studies that have described characteristics of children enrolling in HIV care and treatment programs in sub-Saharan Africa. Even with the remarkable achievement in scaling up of pediatric ART services, still children are enrolled into the program at an older age. The increasing number of pregnant women who are living with HIV receiving ARV prophylaxis, and large number of ART adults patients in our HIV Care and Treatment program could lead to reduced HIV transmission to children and thus affecting the enrollment numbers. However, this is contrary to the expectation that an increase in coverage would result in enrollment of children at younger ages due to observed low access to HIED services, which jeopardize the effectiveness of PMTCT program. A pediatric program in Thailand also reported a high median age at HAART initiation of 7.3 years (IQR: 5.0-9.4) for children [18]. Late enrollment of children may be attributed to weak EID facilities in the country, poor linkage between antenatal care and postnatal care clinics, and lack of skilled human resource in management of pediatric AIDS. Despite WHO recommendations of ART initiation of HIV infected children younger than 12 months, irrespective of the clinical and immunological stage since April 2008, efforts to increase enrollment are yet to yield any remarkable results [19-21]. Several of the facilities providing pediatric ART services in South Africa, however, have reported success in enrolling 53% of eligible children under 2 years old [19]. This indicates that with appropriate strategies, pediatric ART programs in sub-Saharan Africa are capable of enrolling children at much younger ages. Alternatively, with expanding access to ART, our program may be including older children who have previously been not reached, and the efforts to enroll young children will be noted in the years.

Findings show that children enrolled in recent years are more likely to present with late WHO clinical stages of HIV. This indicates that children are more likely to be identified and enrolled into care after they have developed AIDS, which in most cases is later in life. In our setting, several factors are likely associated with late enrollment including poorly accessible diagnostics problems, low health seeking behavior, poor attendance at postnatal clinics, lack of effective community health services and poorly implemented counseling and testing services for HIV, including provider initiated counseling and home-based care testing.

We also found a decrease in the proportion of enrolled children with a history of TB, and a decrease in the percent of those that screened TB positive. This overall decrease in TB burden is similar to trends shown in a cohort of HIV infected children in Uganda, where they observed a reduction of up to 70% of TB cases [22]. However, TB is still a threatening OI among HIV infected patients. Data from Indonesia suggests that up to 27% of all TB cases are seen in children. In this study we found that only 6% of enrolled children had TB, while a cohort of children in Uganda had a TB prevalence of 9.5% [22,23]. Intensive TB screening programs at all CTCs, and ongoing capacity building strategies to support service providers in identification and prompt management of TB cases, are possible factors for the achievements in a reduction of TB cases.

The median HB level increased annually since the start of study. This is contrary to the expectation that children who were enrolled at a late stage of disease and at an older age, were more likely to present with a decline in their clinical status, which could be explained by the effects of age on HB concentration. However, the finding may indicate a ‘survivor bias’ in our dataset, in that those whose who were sicker had died. Anemia has been reported as a prevalent condition in HIV infected children, and it has also been associated with age, CD4 count and clinical or immunological status [22,24,25].

### Our study has several strengths and limitations

This analysis included data collected from a large number of children enrolled in health facilities at different levels of the health care delivery system. Limitations include that only public facilities were included, which may limit generalizability. According to the MDH program progress report, by the end of September 2012, 25% of the HIV patients in DSM were receiving HIV treatment services in private facilities. Given that the provision of ART services is provided free of charge and supported equally in both public and private sectors, and that both sectors use the same national standards, these trends can be inferred to private facilities in Dar es Salaam.

## Conclusions

In conclusion, despite efforts to scale up HIV screening and care and treatment services, children are generally being enrolled at a late stage of HIV disease in the Dar es Salaam region. There is a need to increase the access to HIV Exposed Infant Diagnosis services with corresponding efforts to improve retention and provide timely DBS results to speed up early initiation on ART.
